# Matrisome-Associated Gene Expression Patterns Correlating with TIMP2 in Cancer

**DOI:** 10.1038/s41598-019-56632-3

**Published:** 2019-12-27

**Authors:** David Peeney, Yu Fan, Trinh Nguyen, Daoud Meerzaman, William G. Stetler-Stevenson

**Affiliations:** 1 Extracellular Matrix Pathology Section, Laboratory of Pathology, National Cancer Institute, National Institute of Health, Bethesda, Maryland USA; 2 Computational Genomics and Bioinformatics Group, Center for Biomedical Informatics & Information Technology, National Cancer Institute, National Institute of Health, Rockville, Maryland USA

**Keywords:** Breast cancer, Lung cancer

## Abstract

Remodeling of the extracellular matrix (ECM) to facilitate invasion and metastasis is a universal hallmark of cancer progression. However, a definitive therapeutic target remains to be identified in this tissue compartment. As major modulators of ECM structure and function, matrix metalloproteinases (MMPs) are highly expressed in cancer and have been shown to support tumor progression. MMP enzymatic activity is inhibited by the tissue inhibitor of metalloproteinase (TIMP1–4) family of proteins, suggesting that TIMPs may possess anti-tumor activity. TIMP2 is a promiscuous MMP inhibitor that is ubiquitously expressed in normal tissues. In this study, we address inconsistencies in the literature regarding the role of TIMP2 in tumor progression by analyzing co-expressed genes in tumor vs. normal tissue. Utilizing data from The Cancer Genome Atlas and Genotype-Tissue expression studies, focusing on breast and lung carcinomas, we analyzed the correlation between TIMP2 expression and the transcriptome to identify a list of genes whose expression is highly correlated with TIMP2 in tumor tissues. Bioinformatic analysis of the identified gene list highlights a core of matrix and matrix-associated genes that are of interest as potential modulators of TIMP2 function, thus ECM structure, identifying potential tumor microenvironment biomarkers and/or therapeutic targets for further study.

## Introduction

Matrix metalloproteinases (MMPs) are a family of zinc-dependent endopeptidases that are the major mediators of extracellular matrix (ECM) breakdown and turnover. Humans express 23 MMPs and these play a critical role in tissue development, homeostasis and disease progression. The expression and activity of metzincin proteinases (MMPs and ADAMs), are routinely dysregulated in a number of pathologic conditions, including cancer. Chronic inflammation, oxidative stress as well as infiltration/proliferation of hematopoietic and stromal cells within the tumor environment (TME) are coincident with enhanced expression and activity of MMPs and ADAMs. This enhanced expression is associated with, in addition to amplified ECM proteolysis, increased bioavailability of growth factors and cytokines, inhibition of apoptosis, regulation of tumor vasculature and cellular differentiation^1,2^. The enzymatic activity of metzincin proteinases in the TME is primarily regulated by a family of 4 endogenous proteins called tissue inhibitors of metalloproteinases (TIMPs). Since their original discovery almost three decades ago, several additional biological functions have been ascribed to TIMP family members such as regulation of receptor tyrosine kinase-dependent cell growth and cell migration^3^. TIMP2 is abundantly expressed in normal tissues and demonstrates broad spectrum inhibition of MMPs, as well as forming a high affinity complex with pro-MMP2 independent of the MMP-inhibitory domain^4^. TIMP2 has also been described as interacting with several membrane receptors such as integrin α3β1^5^, membrane-bound MMP14^6^, insulin-like growth factor 1 receptor (IGFR1)^7^ and low density lipoprotein receptor-related protein 1/2 (LRP1/2)^8,9^. In addition, TIMP2 and these putative receptors have been shown to exhibit multiple interactions with components of the ECM.

These findings have led us, and others, to posit that TIMPs are multifunctional proteins and that their specific functions are modulated by the composition of the ECM^10,11^. Thus, TIMP expression in normal tissues, with low levels of metzincin protease expression, may have distinct biologic activities from those of the same TIMP when expressed under pathologic conditions that are frequently associated with high levels of metalloproteinase activity. Recent studies in our lab have shown that TIMP2 is a promising candidate for biological cancer therapy^12,13^. Our working hypothesis is that TIMP2 acts as a homeostatic mediator at the interface between cellular components and ECM in normal tissues. In the evolving TME with both tumor and stromal cells (fibroblast, inflammatory and endothelial cells) that express increasing levels of active MMPs, first the MMP-independent functions and eventually the MMP-inhibitory activity of TIMP2 is overwhelmed. This results in extensive matrix remodeling with concomitant changes in ECM composition and tumor promoting functions. However, the role of TIMP2 in tumorigenesis has been subject to debate with the publication of conflicting reports correlating TIMP2 with either enhanced or poor prognoses.

Recent advances in genetic profiling of tumors led to generation of The Cancer Genome Atlas (TCGA), a comprehensive map of the tumor genome and transcriptome, and the Genotype-Tissue Expression (GTEx) project for the study of tissue-specific gene expression. This extensive trove of data has allowed researchers around the world to interrogate gene expression patterns in normal tissues and diseased states. It is already appreciated that an imbalance between proteases and their endogenous inhibitors contributes to ECM disruption associated with tissue pathology. However, there have been few reports specifically addressing the profile of genes co-expressed with TIMP family members in different cancers and normal tissues using large patient datasets. To enrich our understanding of putative TIMP functions in the TME through identification of target genes for future studies, we examined co-expression patterns specific for each member of the TIMP protease inhibitor family utilizing TCGA and GTEx datasets. This led us to identify previously unrecognized similarities between the patterns of genes that are co-expressed with both TIMP2 and TIMP3. Further interrogation of the TIMP2 co-expressed genes identified highly correlated core matrisome partners that are of interest as modulators for TIMP2 function, specifically in the TME, and are potentially useful either as targets or biomarkers of TME-directed cancer therapies.

## Results

### TIMP expression is altered in cancer tissues

**Figure 1:**
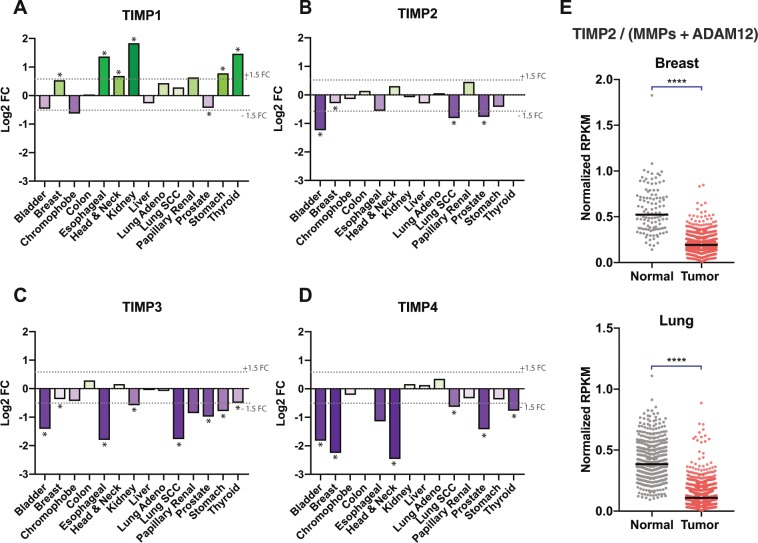
TIMP1–4 display variable levels of differential expression in tumor vs. normal tissue. (**A**–**D**) Differential expression for TIMPs 1–4 in tumor vs. normal tissue were harvested through BioXpress and presented as log2 FC (fold change) following DEseq normalization and analysis of TCGA data, * indicating significance. (**E**) TIMP2 RPKM transcript counts were obtained through the Recount2 platform and normalized using division of TIMP2 RPKM by the sum of MMP and ADAM12 RPKM to show that the TIMP2:metalloproteinase balance is often disrupted tumors. ****p < 0.000, two-tailed t-test.

To start we first highlight changes of TIMP levels in tumors of different organs utilizing several publicly available databases and resources (Table 1). Log2-fold changes in expression of each TIMP were compared between 14 cancer types and corresponding normal tissue from the same organs, all obtained through the BioXpress database, Fig. 1A–D^14^. This analysis demonstrated the variability in TIMP expression levels across various cancer tissues when normalized to controls. As previously reported, TIMP1 expression is frequently up-regulated in cancers (Fig. 1A) and its increased expression has been associated with poor prognoses in numerous studies^11^. In contrast, TIMP3/4 expression levels are significantly lower in several types of cancer. These changes are most pronounced for TIMP3 levels in bladder, lung squamous cell and esophageal carcinomas, as well as bladder, breast, head & neck and prostate for TIMP4, Fig. 1C,D. TIMP2 displays less dynamic, although significant changes in expression at similar cancer sites (bladder, breast, lung squamous cell carcinoma (SCC) and prostate) when compared to normal tissues. Unlike the growing consensus of enhanced TIMP1 expression correlating with poor cancer prognosis, the clinical significance of the absolute levels of TIMP 2–4 expression in general are not as clearly delineated with studies reporting variable prognostic value, as previously reviewed^15^.

**Table 1:** Publicly available databases and resources used in this study.

Resource	Resident Institute	Methods/Data Used
The Cancer Genome Atlas (TCGA)	TCGA Research Network	RNA sequencing data from over 11000 patients
Genotype-Tissue Expression (GTEx)	GTEx Consortium	RNA sequencing data from non-tumor post-mortem tissue
BioXpress	George Washington University Medical Center	DEseq normalization and differential expression of matched tumor vs. normal samples
cBioPortal	Memorial Sloan Kettering Cancer Center	RNA sequencing mRNA co-expression from TCGA
Recount2	Johns Hopkins University	Processed RNA sequencing data from TCGA and GTEx

In order to more clearly understand the significance of TIMP2 expression levels in breast and lung carcinomas, we assessed the levels of TIMP2 in relation to metzincin targets (MMPs and ADAM12) by simple division of the TIMP2 RPKM (Reads Per Kilobase of transcript, per Million mapped reads) by that of MMPs and ADAM12 (sum of the MMP family and ADAM12 RPKM). This analysis highlights a significant reduction in the ratio of TIMP2 versus metalloprotease RPKM in these cancer types when compared with normal breast and lung tissues, Fig. 1E. In accordance with our working hypothesis, the results suggest that the expression of these metzincin protease targets overwhelms the already low levels of TIMP2 in these pathologic tissues, which would result in a reduction of non-protease associated TIMP2 activity. We find that, in lung and breast carcinomas versus normal tissue, the balance between TIMP2 and these known molecular targets is clearly shifted in favor of metalloproteinase expression in tumor tissues (Fig. 1E).

### TIMP2 displays a unique gene co-expression profile in carcinomas

**Figure 2:**
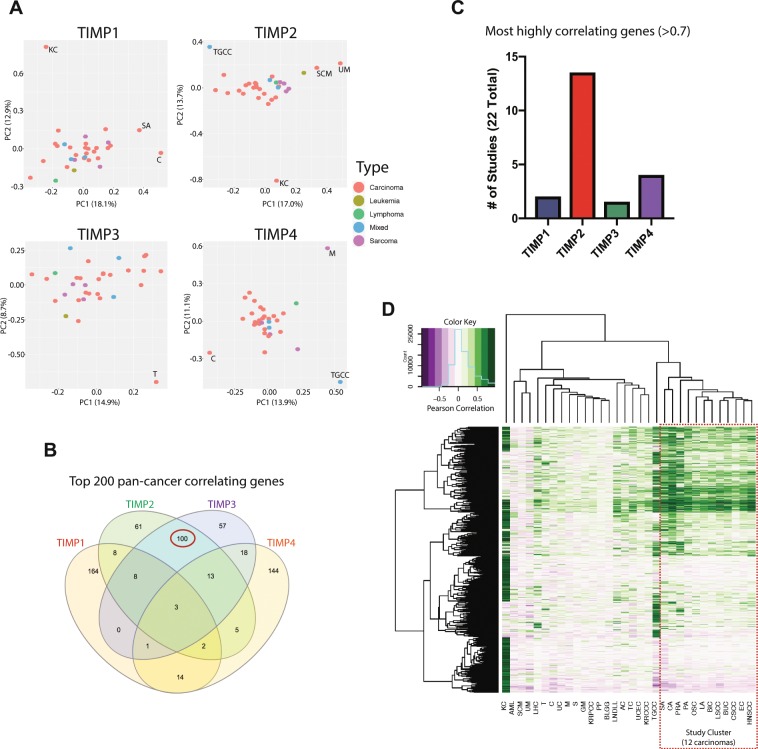
TIMP2 displays a unique co-expression profile in carcinomas. (**A**) TIMP1–4 Pearson’s correlation with the whole transcriptome across the TCGA dataset (32 studies) were collected through cBioportal and principle component analysis was performed on the full data. (**B**) Venn diagram depicting the similarity/dissimilarity between TIMP1–4 in their co-expression profiles. (**C**) TIMP2 displays, in general, the most highly correlating (>0.7) genes in carcinomas in comparison to other members of the TIMP family. (**D**) Hierarchical clustering performed on Pearson’s correlating genes (>0.4 correlation) to highlight a study cluster of 12 carcinomas that display similar TIMP2 co-expression patterns within the TCGA dataset. Abbreviations; C: Cholangiocarcinoma, KC: Kidney Chromophobe, M: Mesothelioma, SCM: Skin Cutaneous Melanoma, T: Thymoma, TGCC: Testicular Germ Cell Cancer, UM: Uveal Melanoma.

In order to initially examine possible relationships between individual TIMP family members and the whole transcriptome based on expression data, we used cBioPortal to harvest Pearson’s correlation scores from 32 TCGA RNA-Seq studies. We then performed principle component analysis (PCA) on correlation scores to gauge the similarity or differences for each TIMP co-expression profile amongst all cancer types within TGCA study repertoire, Fig. 2A. In the TIMP2 and TIMP4 co-expression profiles we observed distinct clustering for the carcinomas. Whereas the TIMP1 and TIMP3 co-expressed gene profiles from cancers of various origins were more interspersed with less apparent grouping of any specific cancer type (carcinoma, sarcoma, hematologic). However, distinct outliers were observed in the co-expression pattern irrespective of the TIMP (e.g. kidney chromophobe cancer (KC) in the TIMP1 and TIMP2 co-expression profiles). It is well documented that TIMP4 gene expression is generally quantitatively lower and more tissue restricted than TIMP2^15^ (Supplementary Tables I and II), thus focusing our study further on TIMP2 and coinciding with our interest in identification of potential modifiers of TIMP2 function.

Following comparison of the pan-cancer correlating genes for each individual TIMP co-expression profile, Fig. 2B, we noted that TIMP2 and TIMP3 were highly similar, sharing 50% of their top 200 correlating genes. Whereas TIMP1/4 were much more distinct from the other family members (~75% unique identity) in their individual co-expression patterns. The observation that, independent of cancer subtype, TIMP2 and TIMP3 exhibit a close similarity in the pattern of co-expressed genes (50% shared identity) implies a potential and previously unappreciated functional parallelism may exist between these co-expressed gene sets that may extend beyond targeting of protease activity. What identifies TIMP2 as the more interesting candidate over TIMP3 is that TIMP2 generally displays the largest number of highly correlating genes (Pearson’s correlation >0.7) across the TIMP family in carcinomas, Fig. 2C. Specific numbers of highly correlating genes for each study are supplied in Supplementary Table III. A pan-cancer hierarchical clustering analysis of Pearson’s scores (containing only genes that display at least −/+0.4 correlation for any one cancer sub-type) for TIMP2 correlating genes highlights a study cluster of 12 carcinomas that display highly similar co-expression profiles for TIMP2, Fig. 2D. This suggests that if any of these co-expressed genes modulate TIMP2 function, then it is a common occurrence in carcinomas. To examine this potential effect in more detail we further focused our study to the co-expression profiles for TIMP2 in normal and carcinomatous lung and breast tissues.

### TIMP2 and protease targets exhibit a distinct expression pattern in tumors

**Figure 3:**
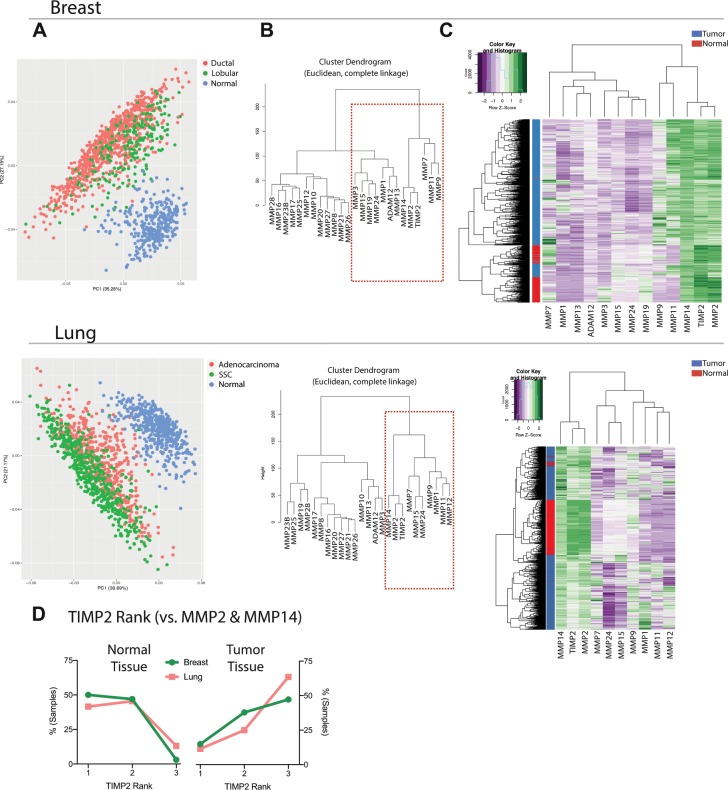
TIMP2 and it’s known molecular targets exhibit a distinct expression pattern in tumors. (**A**) Principle component analysis of Recount2 harvested breast and lung TCGA (tumor and normal tissue) and GTEx (normal tissue) log2 RNA sequencing data (RPKM) depicts unique expression profiles for tumor tissue with regards to the 334 genes highlighted as co-expressed with TIMP2 in tumors. (**B**) Cluster dendrograms for TIMP2, ADAM12 and MMP expression in pooled TCGA and GTEx data highlights gene clusters that show appreciable tissue expression for further analysis (identified by the red dashed box). (**C**) Hierarchical clustering of previously highlighted genes of interest (identified by the red dashed box indicating appreciable levels of expression in Figure 3B) suggests that the TIMP:MMP/ADAM12 balance in tumors is shifted in favor of metalloproteinase activity. (**D**) Expression rank analysis of TIMP2, MMP2 and MMP14 expression (highest expression = Rank 1, lowest expression = Rank 3) from individual normal and tumor tissue samples reveals that TIMP2 expression levels are generally lowest of these three genes in tumor tissue. Abbreviations; SCC: squamous cell carcinoma.

Our earlier analysis of TIMP2 co-expression profiles identified genes that correlate with TIMP2 in cancers, highlighting a need to understand the TIMP2 co-expressed genes in normal tissues. To do this, we first identified a list of 334 genes of interest (GOI) consisting of 300 genes that consistently display a positive correlation with TIMP2 across all cancers types, plus 34 genes we identified as TIMP2 interacting partners, such as the metzincin proteases (MMPs and ADAM12), as well as putative TIMP2 binding partners LRP1/2, extracellular Src kinase, integrin α3β1, and IGFR1 (listed in Supplementary Table IV). We then harvested normalized expression data for the GOI cohort from lung and breast TCGA (tumor and normal tissue) and GTEx (post-mortem normal tissue) studies using Recount2, Fig. 3. We focused specifically on lung and breast carcinomas for several reasons. First, these are common cancers that are within our earlier identified study cluster (Fig. 2D) with significant impact on the global patient population^16^ and prior studies have suggested a correlation between TIMP2 expression levels and prognostic outcome in these cancer populations^17–19^. In addition, earlier work in our laboratory suggests that TIMP2 delivers significant therapeutic benefits in murine lung and breast cancer models^12,20,21^.

PCA of the normalized gene counts for the 334 GOI harvested from Recount2 clearly distinguish breast and lung carcinoma from their normal tissue counterparts, Fig. 3A. However, upon closer inspection these data reveal no clear demarcation between the major subtypes of breast (ductal vs. lobular) or lung cancer (adenocarcinoma vs. squamous cell carcinoma). Previous studies clearly discriminate between gene expression profiles of these carcinoma subtypes^22,23^. The failure of TIMP2 co-expressed gene patterns to discriminate lobular vs. ductal breast cancers, or squamous vs. adenocarcinomas in the lung may suggest that the 334 GOI profile reflects the host response in the tumor microenvironment (TME) rather than tumor autonomous gene expression patterns. Although this explanation awaits future refined analysis of specific tumor compartments (malignant vs. TME), these data could serve as a starting point to reveal potential new targets in the TME for therapeutic intervention that may not be as susceptible to the development of drug resistance or tolerance due to genetic shifts in the tumor cell population.

We then examined expression profiles from the TIMP2 GOI using defined subsets. The first subset of this GOI list focused exclusively on the metzincin protease targets of TIMP2, the MMPs and ADAM12 genes, shortening these lists based on expression level using cluster dendrograms, Fig. 3B. Hierarchical clustering analysis of these shortened gene lists clearly segregates normal tissue from malignant tissue based on the expression levels of TIMP2 and its protease targets, Fig. 3C. These findings suggest that in the TME the dominant biologic function for TIMP2 may be skewed towards metalloproteinase inhibitory activity. An interesting observation is that in the breast carcinoma data MMP7 expression was elevated in a subset of tumor-matched normal tissues (grossly normal tissue obtained at the time of tumor resection) within TCGA dataset. This suggests a potential field effect for the development of cancer and thus associated risk-factors, highlighting the potential utility of MMP7 as a biomarker, as previously suggested for other diseases^24^. It is also of note that the separate tumor groups, identified in Fig. 3C, do not specifically align with gross tumor classifications and may be more indicative of other, previously un-appreciated, cancer sub-types. We consistently observed a significant correlation in the expression of the well-known TIMP2, MMP2 and MMP14 axis^6^. Whilst these data are generally in agreement with the proposed dominant function of TIMP2 as a protease inhibitor, this correlation also suggests another major function of TIMP2 may be altered; namely its well-studied, counter-intuitive role in the cell surface activation of pro-MMP2 via formation of a trimolecular complex with MMP14^6^. We found that tumor associated TIMP2 expression levels in comparison to MMP2 and MMP14 are consistently the lowest of the three genes, whereas in normal tissues TIMP2 is predominant, Fig. 3D. These findings are consistent with the reported increase in MMP2 activation in tumor tissue^1^, as well as supportive of our hypothesis that the MMP-independent functions of TIMP2 are dominant in normal tissues.

### Genes associated with the matrisome and mesenchymal cell lineages acquire a positive correlation with TIMP2 in tumors

**Figure 4:**
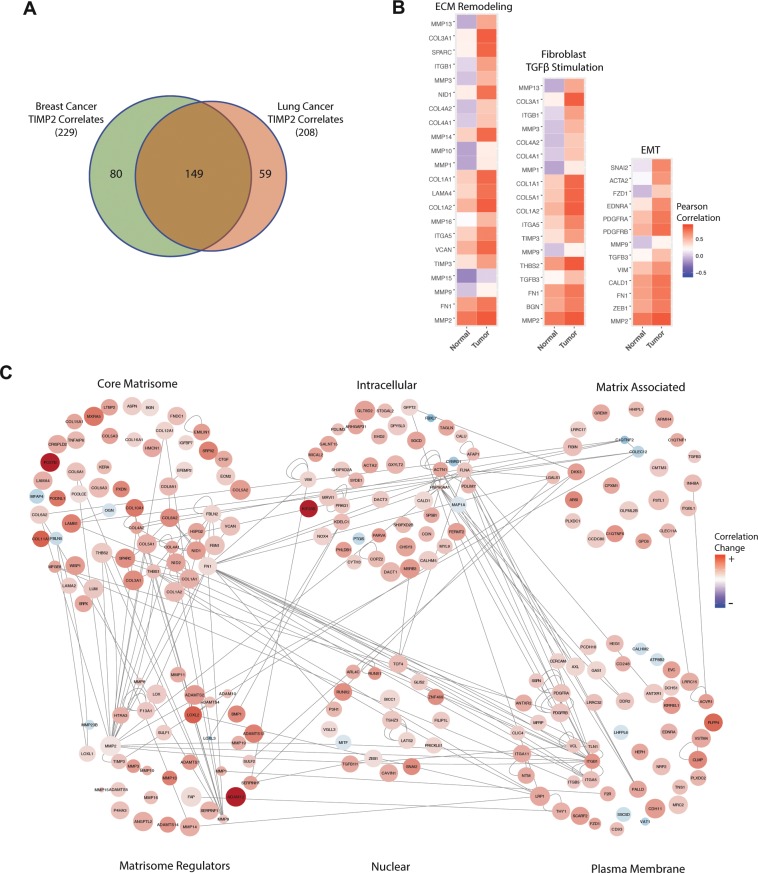
Genes that acquire a positive correlation with TIMP2 in breast/lung tumors are related to the matrisome and a mesenchymal phenotype. (**A**) Venn diagram of significant tumor specific TIMP2 correlating genes between breast and lung tissue. (**B**) Pearson’s correlation heatmap for genes associated with ECM remodeling, TGFβ stimulation of fibroblasts and epithelial-mesenchymal transition (EMT) (adapted from pathway analysis using MetaCore). (**C**) Cytoscape was used to visualize genes that acquire a positive correlation with TIMP2 in tumor tissue as gene ontology networks, with nodes and their color used to depict genes and their correlation change in tumor vs. normal tissue and node size used to depict the correlation score in tumor tissue (no correlation = small node, high correlation = large node). Edges depict reported interactions between nodes (harvested from BioGRID).

In an effort to gauge which set of genes acquire an altered correlation with TIMP2 in tumor tissues, we used the *r.test()* function of the package *psych* in R to test the difference between two sets of independent correlations following calculation of the TIMP2:GeneX correlation scores (Pearson’s) within tumor and normal tissue^25^. We used Bonferroni correction to adjust the p-values, highlighting sets of 229 and 208 genes (adjusted p-value < 0.05) from breast and lung adenocarcinomas, respectively, with a significant overlap of 149 genes between these carcinomas (>60%), as shown in Fig. 4A. The top 10 significant genes from each set are shown in Table 2 (full tables in Supplementary Tables V & VI). These results highlight a substantially significant co-expression signature in which many of the highly co-expressed genes are exclusive to lung tissue. In contrast, all of the top 10 correlating genes from breast tumor tissue were also significant in lung tumor tissue (Table 2). From Ingenuity® Core Analysis we also identified upstream regulators of the significant genes for both lung and breast carcinomas, shown in Table 3 (& Supplementary Tables VII & VIII), highlighting potential drivers of this co-expression profile such as TGFβ and WNT3A. MetaCore™ Pathway Analysis generates broader pathway designations than Ingenuity® and emphasized a number of molecular pathways that are modulated by members of the significant gene lists (Supplementary Tables IX & X). These data were used to generate heatmaps delineating alterations in Pearson’s correlation with genes and their associated pathways using the breast cancer data set as an example, Fig. 4B. Using reference databases (Ingenuity® and the Matrisome Project^26^) and manual designations, significant genes from the breast dataset were assigned to one of 6 major ontologies (core matrisome, matrix regulators, matrix associated, plasma membrane, intracellular and nuclear). Cytoscape was used to visualize changes in correlation, displayed as nodes grouped into their designated ontologies, with edges depicting defined interactions (physical and genetic) between genes, harvested from BioGRID^27^ (Fig. 4C). This analysis highlights the interconnectivity of the TIMP2 correlating genes, providing further evidence that these cancer-associated changes in gene co-expression share the same drivers.

**Table 2:** Significant genes (Bonferroni correction < 0.05) that display the largest changes in Pearson’s correlation in breast and lung tissue samples.

Top genes that acquire a positive correlation with TIMP2 in tumors									
Breast					Lung				
Gene	Cor. Normal	Cor. Tumor	Cor. Δ	Lung?	Gene	Cor. Normal	Cor. Tumor	Cor. Δ	Breast?
POSTN	−0.09	0.78	0.87		CSF1R	−0.25	0.61	0.86	
ADAM12	−0.05	0.82	0.87		WISP1	−0.23	0.49	0.73	
KIF26B	−0.16	0.71	0.87		GGT5	−0.13	0.58	0.71	
LOXL2	−0.13	0.69	0.82		MICAL2	−0.09	0.61	0.70	
COL11A1	−0.16	0.65	0.81		KIF26B	−0.16	0.54	0.69	
PLPP4	−0.03	0.74	0.77		ZNF469	−0.16	0.51	0.67	
PODNL1	−0.06	0.64	0.70		FAM198B	−0.08	0.58	0.67	
MXRA5	0.11	0.80	0.69		COL6A3	0.09	0.76	0.67	
COL10A1	0.04	0.72	0.68		MMP9	−0.26	0.39	0.65	
ADAMTS12	0.11	0.75	0.64		COL11A1	−0.03	0.62	0.65	

Final column indicates whether the correlated gene is also significant in the parallel tissue data set.

**Table 3:** A selection of top potential upstream regulators from the significant genes identified, acquired using Ingenuity Pathway Analysis.

Upstream regulators of TIMP2 correlates			
Regulator	Molecule Type	Gene Hits Breast Lung	
TGFB1	Growth Factor	98	89
Alpha catenin	Membrane Receptor	32	31
HRAS	Enzyme	51	41
TWIST1	Transcription Regulator	27	27
WNT3A	Cytokine	28	30
ITGB1	Membrane Receptor	26	23
ERBB2	Kinase	53	44
TGFB2	Growth Factor	21	19

Potential upstream signaling and transcription factors that have been shown to modulate the genes of interest identified as significant following r.test() analysis. Gene hits are the number of significant genes that can be regulated by the identified regulator.

**Figure 5:**
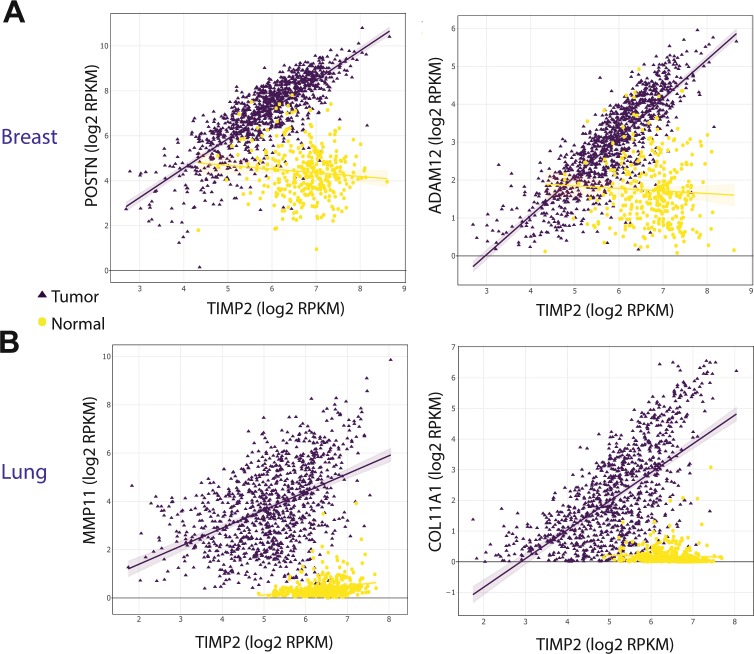
Pairwise comparison of linear regression slopes for TIMP2 versus GeneX. Example linear regressions for TIMP2 versus highly significant genes across both analyses in breast (**B**) and lung (**C**) tissue.

To support our findings from the comparison of Pearson’s coefficients (r.test) shown in Fig. 4, we also performed linear regression on the TIMP2:GeneX relationships and compared the slopes of fitted lines using the *lsmeans* R package (Supplementary Tables XI & XII). Alignment of this data with the r.test results reduced the significant gene hits to 163 and 131 for breast and lung carcinomas, respectively (Supplementary Tables XIII & XIV). Of these gene hits we identified 77 highly significant genes that display an altered relationship with TIMP2 expression in both breast and lung cancer, a selection of which are shown in Table 4. This list represents genes that possess the same or similar ontologies to TIMP2 and exhibit a significantly altered correlation with TIMP2 in tumors that includes metzincin family proteases (ADAM12, MMP11, MMP14), genes associated with increased matrix deposition (collagens I, III. IV, etc.) and also genes associated with stromal activation (FAP), metastasis and pre-metastatic niche formation (FN1, SPARC and POSTN). Whilst many of these genes may acquire a tumor-associated increase in expression irrelevant of TIMP2, it is likely that a number of these genes result in an alteration of TIMP2 activities within developing tumors that contributes to ECM dysregulation. Using a pairwise comparison of linear regression in normal and tumor tissue, we highlight multiple genes for future investigation into their relationship with TIMP2 (Fig. 5). Periostin (POSTN) is a matrix-associated protein that functions as an adaptor/modulator of extracellular interactions^28^, and its expression has been extensively linked to many aspects of carcinogenesis including premetastatic niche formation, invasion and proliferation^29,30^. Interestingly, TIMP2 and POSTN have been described as having a shared distribution during bone development^31^. More recently, we demonstrated that stromal POSTN distribution in metastatic breast tumors of the lung is dramatically altered following TIMP2 treatment^21^. MMP11 and ADAM12 are molecular targets for TIMP2 that are routinely over-expressed in cancers^32,33^. ADAM12 exists in a membrane-bound form (ADAM12-L) and soluble form (ADAM12-S), with ADAM12-L possessing a cytoplasmic tail that interacts with Src homology domains of intracellular proteins. Although TIMP2 has been shown to associate with ADAM12-S, the affinity of the TIMP2:ADAM12-L complex is unknown^34^. The expression of ADAM12 has been shown to localize to tumor vasculature and exert regulatory control over local angiogenesis^33^. Finally, high expression of the α1 chain of collagen XI (COL11A1) is associated with a range of diseases, in addition to an almost uniform high expression in human tumors^35^. It remains to be shown how the altered expression of these targets specifically affects TIMP2 biological activity, either directly or indirectly. We propose that future studies into TIMP2 activity in the TME may reveal potential avenues for the use of TIMP2, or similar biological agents, as a cancer biotherapeutic that may offer viable treatment options in combination with other directed anti-tumor therapies.

**Table 4:** A selection of significant genes from both analyses that occur in both breast and lung datasets.

Gene	Lung Slope Δ	Breast Slope Δ	Ontology
COL10A1	−0.34	−0.65	Core Matrisome
COL11A1	−0.30	−0.48	Core Matrisome
COL1A1	−7.82	−9.42	Core Matrisome
COL1A2	−3.82	−6.16	Core Matrisome
COL3A1	−7.32	−9.75	Core Matrisome
COL4A1	−0.29	−0.41	Core Matrisome
COL5A1	−0.61	−0.56	Core Matrisome
COL6A3	−0.66	−0.65	Core Matrisome
COL8A2	−0.10	−0.19	Core Matrisome
FN1	−2.39	−3.62	Core Matrisome
LAMA2	0.06	0.04	Core Matrisome
POSTN	−0.84	−3.24	Core Matrisome
SPARC	−2.82	−6.02	Core Matrisome
ADAM12	−0.05	−0.16	Matrix Regulators
ADAMTS12	−0.06	−0.05	Matrix Regulators
FAP	−0.05	−0.08	Matrix Regulators
MMP11	−0.90	−1.02	Matrix Regulators
MMP14	−0.87	−0.92	Matrix Regulators
RUNX1	−0.04	−0.07	Nuclear
RUNX2	−0.01	−0.02	Nuclear

Table depicting genes that display high significance in both r.test (Pearson’s correlation) and lstrends (linear regression slopes) analyses in both breast and lung cancer.

## Discussion

Over the past few decades, our understanding and appreciation of the characteristics and hallmarks of cancer progression have greatly expanded however, in these descriptions, dysregulation of ECM turnover and function are often overlooked. In spite of this, the ECM is considered as a critical regulator of all aspects of cell and tissue biology including development^36^, homeostasis^37^, cell differentiation^38,39^, wound healing^40^ and cell motility^41^. As major regulators of ECM proteolysis and turnover, MMPs have a key role in all of the aforementioned biological processes. In turn, as major regulators of metalloproteinase proteolysis, TIMPs are vital regulators of ECM stability, structure and composition. Aberrant MMP activity has long been implicated in carcinogenesis and metastasis^42^, an observation that led to the hasty implementation of failed clinical trials with MMP inhibitors. Since this failure, diverse functions of the metzincin family of metalloproteinases have been identified, both protease-dependent^43^ and -independent^44^. Analogous to the metzincin proteases, diverse functions have been unveiled for the TIMP family of proteins, both metzincin inhibitory-dependent and -independent^45^.

Of the TIMPs, TIMP2 is most widely expressed and observed in all normal tissues, with TIMP4 being the most restricted in its tissue expression^15^. This ubiquitous expression supports the notion that TIMP2 displays homeostatic functions independent of its MMP-inhibitory activity. Interestingly, TIMP2-deficient mice do not exhibit any gross morphological or phenotypic effects in health^46^. However, under conditions of pathological-induced stress, such as tumor development, we have shown that TIMP2-deficient mice show an unfavorable phenotype^12^. Furthermore, TIMP2 deficiencies are associated with abnormal motor function and cognitive dysfunction^47,48^. In addition, we have shown that administration of TIMP2, both directed and systemic, can reduce primary tumor burden, normalize tumor-associated vasculature, reduce infiltration of myeloid-derived suppressor cells and inhibit metastatic niche gene signatures at metastatic target sites^12,13^. Distinct from these findings, there have been conflicting reports linking TIMP2 biological activity^49,50^ and expression^51^ to poorer prognoses.

Although the balance between TIMPs and MMPs has been described as a critical indicator of ECM proteolysis, it should be appreciated that ECM composition is regulated by highly complex mechanisms that are influenced by stromal cell activity and infiltration, inflammatory signaling and other chemical/physical cues within the tissue microenvironment^52^. The balance between TIMPs and MMPs has been briefly investigated in some instances however we sought to gain an understanding of TIMP2 expression relative to the whole transcriptome in health and malignancy.

Our choice to utilize Pearson’s correlation and linear regression to assess the relationship between TIMPs and *GeneX* reflects our belief that classical methods of differential expression are not sufficient for understanding dynamic changes in matrix biology through health and disease. Application of these methods to interrogate the balance between ECM structural components, regulators (TIMPs and MMPs) and associated genes could potentially reveal biological consequences that are not evident when using differential expression analysis. We observed a significant overlap in the co-expression profile of TIMP2 and TIMP3, in addition to the more dynamic scale for down-regulation of TIMP3 expression versus TIMP2 in tumors. This observation that, independent of cancer subtype, TIMP2 and TIMP3 exhibit a close similarity in the pattern of co-expressed genes (50% shared identity) reveals an unexpected parallel functionality for these TIMPs and that the co-expressed gene sets may extend functional effects beyond direct protease activity. However, further consideration of these potential effects is beyond the scope of the current study and awaits future examination utilizing specific knockdown of putative upstream/downstream gene targets.

As noted earlier, previous reports have highlighted that TIMP2 inhibits tumor-associated angiogenesis and leads to a ‘normalization’ of tumor vasculature^12,13^. Similar to TIMP2, TIMP3 has been identified as a critical regulator of numerous microvascular endothelial cell functions, and also as a critical regulator of pericyte function required for proper management of vascular permeability through multiple MMP-independent and -dependent functions^53,54^. This highlights a level of redundancy between TIMP2 and TIMP3 activities and raises the intriguing idea that combinatorial administration of TIMP2 and TIMP3 to tumors may augment previous anti-tumor findings observed with TIMP2.

With regards to carcinomas, TIMP2 displays a strong correlation with a large number of genes, many of which belong to components of the matrisome and supporting our idea that TIMP2 function may be modulated at a post-transcriptional level in tumors. Significantly, we observed that the 334 GOI subset of TIMP2 co-expressed genes readily distinguish breast and lung carcinomas from normal controls, however, it is somewhat surprising that this gene subset did not differentiate breast or lung carcinoma subtypes (ductal vs. lobular; adenocarcinoma vs. squamous) that are easily differentiated by conventional gene expression profiling^22,23^. We posit this gene list potentially reflects a host response of stromal origin, autonomous of direct tumor cell expression. Thus, this 334 GOI cohort may reflect novel tumor microenvironment gene targets and/or biomarkers.

The matrisome is an ensemble of genes that make up the ECM proteome. This consists of structural (core) components such as collagens and glycoproteins, matrisome-regulators (including metalloproteinases and TIMPs) and a large number of matrisome-associated genes as defined by the Matrisome Project^26^. In addition, putative targets for TIMP2 include non-protease targets such as integrin α3β1, IGF1R and LRP1/2^55–58^. These non-protease targets also exhibit extensive interactions with components of the ECM and/or with each other, collectively this extensive network of putative TIMP2 targets/partners further strengthens our hypothesis that altered ECM composition determines TIMP2 biological functions.

It is well documented that TIMP2 is a broad spectrum inhibitor of MMPs^59^ in addition to ADAM12^34^, with the affinity of these inhibitory complexes ranging from sub-fM to low-nM^60,61^. Surprisingly, there have been few, if any, studies into the fate of these TIMP:MMP/ADAM12 complexes or whether combinations of these impart modulatory effects on TIMP2 function. To add further complexity to the system, many of TIMP2’s molecular targets can be proteolytically cleaved by metalloproteinase activity^62–64^. We focused our in-depth analyses on breast and lung tumors, since we have found that TIMP2 can deliver therapeutic effects in murine models of these cancers and that they formed the nucleus of our identified study cluster. With regards to TIMP2:MMP expression, breast tumors seemed to be more consistent with an almost ubiquitous increase in MMP11 and a smaller number of patients exhibiting high MMP9 expression. Lung tumors, on the other hand, displayed a more inconsistent pattern of MMP expression, with subsets of tumors showing high levels of different MMPs such as MMP14, MMP1, MMP7, MM9 and MMP12. It would be of interest to assess whether any of the observed patterns of MMP expression are associated with clinical features (such as tobacco use, radon exposure, occupational hazard, etc.) or genetically distinct tumor subtypes defined by specific driver mutations. Lung tumor tissue also displayed a unique profile of highly significant TIMP2 co-expressed genes (Table 2), the potential causes and consequences of which are likely related to a complex disease etiology.

Through our understanding of MMP and TIMP expression in healthy versus neoplastic tissues, we may be able to elucidate mechanisms by which metalloproteases can alter TIMP functions. Interestingly, MMP2 and TIMP2 expression remained highly correlated in both health and disease. MMP2 is secreted as a zymogen (pro-MMP2) which forms a high affinity complex with TIMP2 via its C-terminal hemopexin domain and the non-inhibitory C-terminal of TIMP2^4^. Counterintuitively, the activation of MMP2 is largely dependent on its endogenous inhibitor TIMP2^46^. This activation is mediated via a tri-molecular complex between TIMP2:pro-MMP2 complex and membrane-tethered MMP14, a complex that allows a free MMP14 to cleave the pro-domain of MMP2 and leads to the release of active MMP2. In tumor tissue, the balance between TIMP2, MMP2 and MMP14 is tipped in favor of MMP14 and MMP2 suggesting that the levels of MMP2 activation in tumor tissue are increased. MMP14 demonstrates weak proteolytic activity against collagen IV, however MMP2 is a potent collagen IV protease that supports invasion into the collagen IV rich basement membrane^65^.

Interestingly, it has recently been described that TIMPless (TIMP family deficient) fibroblasts acquire cancer-associated fibroblast-like features^66^ that, in combination with our study, suggests that there is a yet-to-be determined regulatory relationship between MMPs, TIMPs and fibroblast activity. Indeed, expression of both MMPs and TIMPs are most closely associated with cells from the stromal compartment, including both resident and infiltrating cells^1^. On this premise one might anticipate that TIMP expression should increase in tumors, particularly those that are highly fibrotic in nature such as breast, lung and pancreatic carcinoma. As described above, our analysis found that the genes which acquire a strong correlation with TIMP2 are associated with the extracellular matrix and cells of a mesenchymal lineage and surprisingly do not differentiate carcinoma subtypes (Fig. 3A). The implications and downstream significance of which are currently under investigation. The research described here is limited by the fact that expression data is used as the sole source of analysis. Regulation of extracellular protease activity is broad and complex, including but not limited to: transcriptional expression, pro-enzyme activation, localization (intracellular, matrix bound, cell surface bound), presence of binding partners, cleavage and post-translational modifications. Metalloprotease researchers are currently limited in their ability to broadly assess metzincin protease regulation and activity *in situ*, highlighting the importance of transcriptomic data to identify target groups for in depth functional analyses. This study highlights a number of matrisome-associated genes and regulators for future investigations into their role in the regulation of TIMP2 biological functions.We propose that targeting the imbalance between MMPs and their inhibitors to restore the enhanced TIMP2 expression observed in normal tissues may prove a valuable treatment option for a number of carcinomas.

## Methods

### Differential expression of TIMP family genes in cancer tissue

Log2 fold change expression results for TIMP1–4 were obtained for tumor tissue versus corresponding normal tissue via the BioXpress database^14^ for 14 cancer studies and presented as histograms for visualization purposes. This database uses DESeq to assess differential expression^67^.

### Identification of focused gene list

To support the identification of discrete gene signatures associated with TIMP2 expression in tumors, we generated a list of 334 genes that consistently display a high correlation with TIMP2 in tumors or have the capacity to regulate TIMP2 function. Pearson’s correlation for TIMP2 versus the transcriptome for each TCGA study were harvested using cBioPortal’s co-expression function^68^.

### Harvesting of RNA sequencing data

TCGA and GTEx *RangedSummarizedExperiment (RSE)* gene-level objects were downloaded from the Recount2 (https://jhubiostatistics.shinyapps.io/recount/) project and converted to RPKM (Reads Per Kilobase of transcript per Million mapped reads) using the *getRPKM()* function through the *recount* R package. Legacy universally unique identifiers (UUIDs), which are used as sample IDs in the Recount2 TCGA data, were converted to sample barcodes using the *GenomicDataCommons* R package and manual download of the JSON manifest files from the selected study using the Genomic Data Commons legacy archive (https://portal.gdc.cancer.gov/legacy-archive/). TCGA clinical data was obtained from cBioPortal using the R package *cgdsr* using the *getClinicalData()* function and samples were marked with the appropriate identification depending on tissue of origin and cancer sub-type. Formalin-fixed paraffin-embedded samples and duplicates were removed before further analysis.

### Co-expression analysis

To identify genes that acquire a correlation with TIMP2 specifically in tumors, Recount2 processed TCGA tumor and pooled TCGA/GTEx normal tissue RPKM counts were analyzed to calculate Pearson’s correlation for TIMP2 versus GeneX in normal and tumor tissue. We then used the *r.test()* function of the R package *psych* to perform a z test of the Fisher’s z transformed correlations, divided by the standard error of the difference^25^, to test the difference between two independent (tumor and normal) correlations. Additionally, linear regression using the *lm()* function in R was performed followed by pairwise comparison of linear regression slopes using the *lstrends()* and *pairs()* function of the R package *lsmeans*. Acquired p-values for both analyses were corrected for multiple testing using Bonferroni Correction.

### Visualization of results

*R.test()* significant genes from the breast tissue data were assigned one of six specific gene ontology identifications (core matrisome, matrix regulators, matrix associated, plasma membrane, intracellular and nuclear) based on reference databases (Ingenuity® and the Matrisome Project^26^) and manual definitions. Pathway analysis of significant *r.test()* genes was performed using MetaCore™ and highly significant pathways and gene hits were incorporated into Pearson’s heatmaps using the *ggplot2* package in R. Linear regression plots were generated using the R package *plotly* with 95% confidence intervals.

## Supplementary information

Supplementary Dataset 1

Supplementary Dataset 2

Supplementary Dataset 3

Supplementary Dataset 4

Supplementary Dataset 5

Supplementary Dataset 6

Supplementary Dataset 7

Supplementary Dataset 8

Supplementary Dataset 9

Supplementary Dataset 10

Supplementary Dataset 11

Supplementary Dataset 12

Supplementary Dataset 13

Supplementary Dataset 14

## Data Availability

The authors confirm that the data supporting the findings of this study are available within the article [and/or] its Supplementary Materials.
